# Induction of Murine Macrophage M2 Polarization by Cigarette Smoke Extract via the JAK2/STAT3 Pathway

**DOI:** 10.1371/journal.pone.0107063

**Published:** 2014-09-08

**Authors:** Fengjiao Yuan, Xiao Fu, Hengfei Shi, Guopu Chen, Ping Dong, Weiyun Zhang

**Affiliations:** 1 Jiangsu Key Laboratory of Molecular Medicine, Medical School, Nanjing University, Nanjing, China; 2 The Affiliated Drum Tower Hospital of Nanjing University Medical School, Nanjing, Jiangsu Province, China; The Ohio State University, United States of America

## Abstract

Cigarette smoking is a major pathogenic factor in lung cancer. Macrophages play an important role in host defense and adaptive immunity. These cells display diverse phenotypes for performing different functions. M2 type macrophages usually exhibit immunosuppressive and tumor-promoting characteristics. Although macrophage polarization toward the M2 phenotype has been observed in the lungs of cigarette smokers, the molecular basis of the process remains unclear. In this study, we evaluated the possible mechanisms for the polarization of mouse macrophages that are induced by cigarette smoking (CS) or cigarette smoke extract (CSE). The results showed that exposure to CSE suppressed the production of reactive oxygen species (ROS) and nitric oxide (NO) and down-regulated the phagocytic ability of Ana-1 cells. The CD163 expressions on the surface of macrophages from different sources were significantly increased in *in vivo* and *in vitro* studies. The M1 macrophage cytokines TNF-α, IL-12p40 and enzyme iNOS decreased in the culture supernatant, and their mRNA levels decreased depending on the time and concentration of CSE. In contrast, the M2 phenotype macrophage cytokines IL-10, IL-6, TGF-β1 and TGF-β2 were up-regulated. Moreover, phosphorylation of JAK2 and STAT3 was observed after the Ana-1 cells were treated with CSE. In addition, pretreating the Ana-1 cells with the STAT3 phosphorylation inhibitor WP1066 inhibited the CSE-induced CD163 expression, increased the mRNA level of IL-10 and significantly decreased the mRNA level of IL-12. In conclusion, we demonstrated that the M2 polarization of macrophages induced by CS could be mediated through JAK2/STAT3 pathway activation.

## Introduction

Cigarette smoking (CS) has reached epidemic proportions worldwide and is the most prevalent cause of many serious disorders including respiratory diseases, cancers and other problems related to the kidney, liver, cardiovascular and pancreas [1]–[6]. Lung cancer is the most important cause of death from neoplastic diseases worldwide. Tobacco smoking is well known to be the dominant risk factor for lung cancer in which tumor-associated macrophages participate in the development of the disease. However, the functional role of macrophages in the initiation of lung cancer is still not well understood.

In living organisms, macrophages play a central role in both specific and nonspecific immunity against environmental pollutants, bacteria and cancer. Macrophages attack foreign substances, infectious microbes and cancer cells as well as phagocytose degenerated cells or cellular debris. Macrophages also produce inflammatory factors that participate in various physiological and pathological processes [7]. Macrophages are the most abundant immunocytes in lung tissue. Pulmonary macrophages, called alveolar macrophages, are widely distributed in the lung alveoli and interstitium and are primarily responsible for the lung's defense function.

Heterogeneity and plasticity are important features of macrophages that promote the development of certain disorders [8]. Macrophages may respond to various stimuli by polarizing into different phenotypes [9]. M1 macrophages are classically activated, resulting in important effector functions including the production of superoxide anions, oxygen radicals and reactive nitrogen species such as nitric oxide (NO) produced by inducible nitric oxide synthase (iNOS) [10]. These macrophages also secrete relatively high amounts of pro-inflammatory cytokines (e.g., TNF-α, IL-1, IL-12 and IL-23), regulate antigen presentation to T-cells and stimulate Th1 T-cell function. Furthermore, M1 macrophages express high levels of MHC II and secrete complement factors that facilitate complement-mediated phagocytosis [9]. In contrast, activated M2 macrophages express high levels of the scavenger receptor (SR), the mannose receptor (MR), IL-10 and TGF-β, which primarily enable macrophages to function in tissue remodeling, immune suppression and tumor progression [10]. The deactivation of the M1 polarization pattern is accompanied by up-regulation of the gene expression that is associated with M2 polarization programs [11].

The prognosis of cancer patients has been found to depend on the ratio of M1 and M2 macrophages [12]. The cytotoxic M1 phenotype explains the extended survival of patients with non-small cell lung cancer (NSCLC), suggesting that positive immune responses play a crucial role in preventing NSCLC progression [13]. However, most of these macrophage studies have focused on cancer patients and have only demonstrated a relationship between macrophage polarization and tumor development.

We found that high doses of cigarette smoke extract (CSE) could induce apoptosis in macrophages and that low doses of CSE usually initiated the macrophage's anti-apoptotic program [14]. The objective of this study was to demonstrate that cigarette smoke could stimulate macrophage transformation toward the M2 phenotype and to determine the corresponding mechanism.

## Materials and Methods

### Determination of surface-specific molecules of M2 macrophage phenotype

To estimate the effect of CSE on the macrophage phenotype, specific macrophage molecule markers were detected by flow cytometry using Ana-1 cells. CSE was prepared as previously reported [14]. Ana-1 cells were treated with 2% CSE for 2, 3, 4, 5 and 6 days and were then stained with an FITC-labeled CD163 anti-mouse antibody (AbDSerotec, Oxford, UK) or a FITC-conjugated MHC-II anti-mouse antibody (eBioscience, San Diego, CA) at 4°C for 40 min. The cells were washed 3 times to remove the unbinding antibody and re-suspended in phosphate buffer saline (PBS, pH 7.4) containing 2% paraformaldehyde. The fluorescence of the cells was measured by flow cytometry (FACS Calibur; BD Biosciences Pharmingen; San Jose, CA, USA). Forward and side scatter parameters were used to gate the live cells. At least 10,000 cells were analyzed per sample.

### Assay of intracellular reactive oxygen species (ROS) of Ana-1 cells after CSE treatment

After the Ana-1 cells were exposed to various CSE concentrations (0, 0.5%, 1%, 2% and 4%) for 24 h, the cells were collected and washed with cold PBS and then incubated with 150 µl of 10 µM dichlorofluorescin diacetate (DCFH-DA, Sigma, St. Louis, USA) for 30 min at 37°C in the dark. The cells were then re-suspended in PBS after washing and detected using flow cytometry (FACS Calibur; BD Biosciences Pharmingen; San Jose, CA, USA) with 488-nm excitation and 530-nm emission wavelengths (FL1 channel). A total of 10,000 events were acquired. The results were analyzed using Flowjo7.6.1 software (Tree Star, Ashland, OR, USA). The mean fluorescence intensity (MFI) was used for the ROS analysis.

### Measurement of NO released by Ana-1 after CSE treatment

One hundred microliters per well of cell culture supernatant was incubated with an equal volume of Griess reagent for 10 min after the cells were treated with CSE (0, 0.5%, 1%, 2% and 4%) for 24 h and 48 h. Nitrite production was estimated by comparing the absorbance at 550 nm with a standard curve generated using NaNO_2_.

### Phagocytic assay by FITC-dextran internalization in Ana-1 cells

The macrophage phagocytic ability was measured by the FITC-dextran internalization of cells. Ana-1 cells were cultured with CSE (0, 0.5%, 1%, 2% and 4%) for 24 h and then incubated with FITC-dextran (Sigma, St. Louis, USA, 1 mg/ml in RPMI1640) for 1 h. The cells were detected using a flow cytometer (FACS Calibur; BD Biosciences Pharmingen; San Jose, CA, USA) after terminating the reaction with cold PBS containing 2% fetal bovine serum (FBS) and washing with PBS containing 2% paraformaldehyde.

### Analysis of primary mouse macrophage culture and CD163 marker assay by flow cytometry

This study was approved by the Ethics Committee of Nanjing Drum Tower Hospital. Male BALB/c mice were purchased from Yangzhou Medical Center, maintained in a specific pathogen-free environment in our animal facility. Six mice were sacrificed using the cervical dislocation method under ether anesthesia at 8–10 weeks of age, and their abdominal cavity fluids were collected and immediately centrifuged at 1000 r/min for 10 min to collect cells. Subsequently, all of the cells were re-suspended in RPMI 1640 and added to 24-well plates (4×10^5^ cells/well) for attachment. The non-adherent cells were then removed by washing (thrice) with warm PBS after 4 h of incubation, and the adherent cells were incubated overnight in complete medium to form a macrophage monolayer. The peritoneal macrophages (PMs) were then treated with different CSE concentrations (0.125%, 0.25%, 0.5%, 1% and 2%) for 3 days. CD163^+^ cells were detected by flow cytometry. The culture supernatants were collected and stored at −80°C for the ELISA assay.

Mouse bone marrow-derived macrophages (BMDMs) were prepared as follows: Male BALB/c mice (8–10 weeks old) were sacrificed using the cervical dislocation method under ether anesthesia, and the femurs and tibias of the mice were flushed with ice-cold PBS in a glass culture dish. The bone marrow cells were collected after centrifugation at 1000 r/min for 10 min. The cells were then seeded into 6-well plates (2 ml/well) at a density of 1×10^5^ cells/ml in medium, and recombinant granulocyte-macrophage colony-stimulating factor (GM-CSF, Peprotech) was added to the culture at a final concentration of 20 ng/ml. After the cells were incubated at 37°C in a 5% CO_2_ incubator for 3 days, the plates were centrifuged, 1 ml of supernatant was removed, and 1 ml of fresh medium containing the same concentration of GM-CSF was added. On day 7, the adherent cells were determined to be M1 monocyte-derived macrophages [15]. The cells were treated with different concentrations of CSE (0.125%, 0.25%, 0.5%, 1% and 2%) for another 3 days and were detached from the culture dish using cold PBS with 20 mM EDTA. CD163^+^ cells were detected by flow cytometry. Culture supernatants were collected for the ELISA assay.

BALB/c mice were sacrificed as above. Mouse lung alveolar macrophages were collected by fitting a venous indwelling needle into a mouse trachea, and PBS was injected to wash the lung six consecutive times (0.8 ml/wash). Total bronchial alveolar lavages were collected and washed with PBS. Then, all of the cells were re-suspended in RPMI 1640 and added to 24-well plates (4×10^5^ cells/well) for 4 h of incubation for attachment. The non-adherent cells were removed by washing (thrice) with warm PBS. The adherent cells were incubated overnight in complete medium to form a macrophage monolayer and then treated with different CSE concentrations (0.125%, 0.25%, 0.5%, 1% and 2%) for 3 days. The cells were also analyzed using flow cytometry.

### Animal study and estimation of CD163^+^ lung alveolar macrophages

To investigate *in vivo* cigarette smoke (CS) exposure, 10 male BALB/c mice were randomly divided into 2 groups. Five mice were used for CS treatment, and the other mice were exposed to air as controls. The CS treated mice were placed in a cage (25×30×18 cm) with 2 filter windows. Cigarette smoke was drawn through a narrow hole continuously by a peristaltic pump. Five mice were exposed to 5 cigarettes a day for 6 days. The control mice were concurrently exposed to air. On day 7, the mice were sacrificed using the cervical dislocation method under ether anesthesia, and the lung alveolar macrophages were collected as described above. CD163^+^ macrophages were detected by flow cytometry.

### Detection of mRNA levels of IL-6, IL-10, IL-12, TNF-α, iNOS, TGF-β1 and TGF-β2 by RT-PCR in Ana-1 cells

First, the effects of different CSE doses on macrophage phenotype-related cytokines were estimated. Ana-1 cells were treated with CSE at doses of 0, 2.5%, 1%, 2% and 4 for 48 h and then collected to detect the mRNA levels of IL-6, IL-10, IL-12, TNF-α, iNOS, TGF-β1 and TGF-β2.

The total RNA extraction and PCR were performed as previously described [16]. The primers and expected product length for this experiment are shown in Table 1. The PCR products were detected on a 2% agarose gel containing ethidium bromide and were visualized under UV light. The results are expressed as the intensity relative to the intensity of the housekeeping gene β-actin.

**Table 1:** Primer sequences of investigated genes in RT-PCR analysis.

Gene	Primer sequences	Product length (bp)
β-actin	F 5′–AGGCATCCTGACCCTGAAGTAC-3′	389
	R 5′-TCTTCATGAGGTAGTCTGTCAG-3′	
TNF-a	F 5′-CCACATCTCCCTCCAGAAAA-3′	702
	R 5′-CGGACTCCGCAAAGTCTAAG-3′	
iNOS	F 5′-GTCTTGCAAGCTGATGGTCA-3′	602
	R 5′-GGCCTCAGCTTCTCATTCTG-3′	
IL-12p40	F 5′ –CAACATCAAGAGCAGTAGCAG-3′	303
	R 5′-TACTCCCAGCTGACCTCCAC-3′	
IL-10	F 5′–TACCTGGTAGAAGTGATGCC-3′	251
	R 5′–CATCATGTATGCTTCTATGC-3′	
TGF-β1	F 5′-GGAACTCTACCAGAAATATAGC-3′	143
	R 5′-CCTGTATTCCGTCTCCTTG-3′	
TGF-β2	F 5′-CCGAGCAGCGGATTGAACTG-3′	133
	R 5′-GCGTCTGTCACGTCGAAGG-3′	
IL-6	F 5′-GCCTTCTTGGGACTGATG-3′	391
	R 5′-CTGGCTTTGTCTTTCTTGTTA-3′	

The effects of different CSE treatment times on these cytokines were also measured. Ana-1 cells were treated with 2% CSE for different durations (24 h, 48 h and 72 h), and their cytokine mRNA levels were estimated as described above.

### Protein level estimation of p-STAT3 and p-JAK2 in Ana-1 cells after CSE treatment by Western blotting

Ana-1 cells (2×10^5^/well) were treated with different CSE doses for 1.5 h. The cells were then collected and washed with PBS and lysed with RIPA Reagent (Beyotime Biotech, Shanghai, China) containing phosphatase inhibitor (1%) (Roche, Basel, Switzerland) on ice for 60 min. After centrifugation at 12,000 g/min for 20 min, the supernatants were harvested and used as total cell extracts to detect STAT3/p-STAT3 and JAK2/p-JAK2.

The extracted proteins were subjected to 10% SDS- polyacrylamide gels and transferred onto nitrocellulose membranes. The membranes were blocked with 5% bovine serum albumin (BSA, Roche, Basel, Switzerland) at 37°C for 1 h and probed using primary antibodies of phosphorylated signal transduction and activators of transcription-3 (p-STAT3, Tyr705), phosphorylated janus kinase 2 (p-JAK2, Tyr1007/1008) (Cell Signaling Technology, Beverly, MA, USA), STAT3, JAK2 (Bioworld Technology, Atlanta, Georgia, USA) at 1∶1000 or Histone, β-actin (Abmart, Shanghai, China) at 1∶5000 overnight at 4°C. The membranes were washed 4 times with PBST (PBS containing 0.1% Tween 20) at room temperature and then incubated with horseradish peroxidase-conjugated goat anti-rabbit IgG secondary antibody (1∶5000 dilution) for 1 h at room temperature. The membranes were washed 4 times again in PBST and subsequently developed using an enhanced chemiluminescence Western Blotting Kit (Beyotime Biotech, Shanghai, China). Densitometry was performed using the software iPP 6.0. The p-STAT3 and p-JAK2 values were normalized by the total STAT3 and JAK2 values.

### Detection of cytokine levels secreted from Ana-1 cells, PMs and BMDMs by ELISA

The culture supernatants were collected from the CSE-treated Ana-1 cells, PMs and BMDMs, and the protein levels of the cytokines IL-10, IL-12, TNF-a, TGF-β1 and TGF-β2 were determined using ELISA kits (R&D Systems, Minneapolis, MN, USA) according to the manufacturer's instructions.

### Effect of p-STAT3 inhibitor WP1066 on CSE-treated Ana-1 cells

Ana-1 cells were cultured in the presence or absence of the STAT3 phosphorylation inhibitor WP1066 (3 µM) for 1 h prior to 1% CSE treatment for 48 h. The vehicle DMSO was used as a control. The IL-12 and IL-10 mRNA expressions of the cells were estimated by quantitative real-time RT-PCR using an ABI7300 Real Time PCR System (Applied Biosystem, Foster City, USA). The amplification conditions were as follows: 50°C for 2 min, 95°C for 10 min, 40 cycles of 95°C for 15 s, 58°C for 30 s and 72°C for 30 s. The PCR specificity was confirmed by performing a melting curve analysis for each data point. 2^−△△Ct^ was used in the mRNA quantitation.

Ana-1 cells were pipetted onto coverslips that had been previously pretreated with poly-D-lysine (Sigma, Missouri, USA) and placed into 6-well microplates. The cells were incubated for 24 h and treated as described above. The coverslips containing the Ana-1 cells were washed 3 times with PBS followed by incubation with cold 4% paraformaldehyde for 20 min. The coverslips were washed 3 times and then blocked using 5% BSA for 1 h. The blocking solution was then removed, and the cells were incubated with an FITC-labeled CD163 anti-mouse antibody (AbDSerotec, Oxford, UK) for 1 h more at room temperature in the dark. The cells were washed twice with PBS (pH 7.4), stained with DAPI (0.5 µg/ml) (Roche, Germany) and then mounted. The cells were examined using a confocal laser scanning microscope (Olympus FluoView, Japan).

### Statistical analysis

The data were expressed as the mean ± SD and subjected to one-way ANOVA followed by Tukey's test. The differences between the mean values were considered significant when p<0.05.

## Results

### Changes in CD163 and MHC-II expressions on the surface of CSE-treated Ana-1 cells

**Figure 1:**
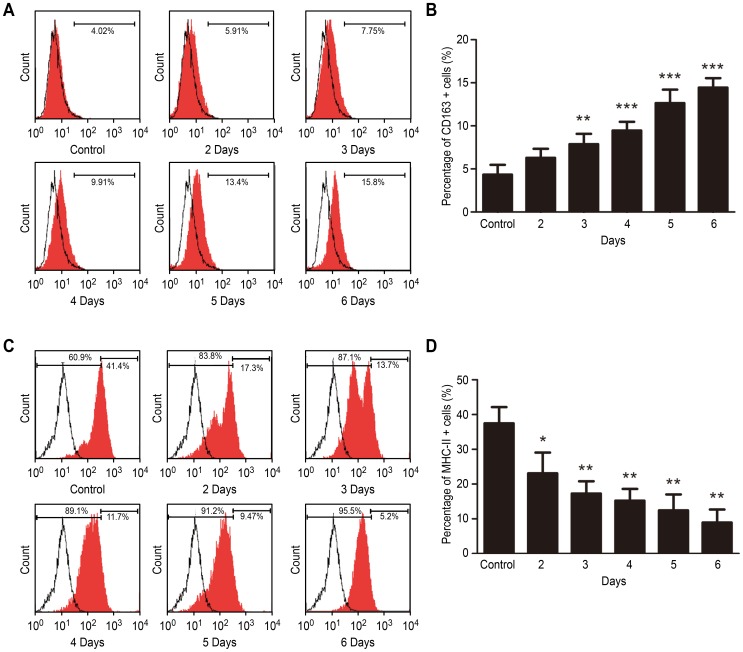
Surface antigen expression assay after mouse macrophages were treated with CSE for different time. **A, B** Effect of CSE on the expression of CD163 in Ana-1 cells. **C, D** Effect of CSE on the expression of MHC II in Ana-1 cells. n = 4. *p<0.05, **p<0.01, ***p<0.001.

M2 macrophages usually express a high level of the cell surface scavenger receptor (the CD163 marker) and a low level of MHC-II molecules. To evaluate whether the CSE stimulated Ana-1 cell polarization toward the M2 phenotype, the CD163 and MHC-II expression levels were first assessed by flow cytometry. Figure 1A shows that after the Ana-1 cells were treated with 2% CSE, the CD163 expression increased significantly. In contrast, the MHC- II expression was down-regulated after the Ana-1 cells were treated with CSE relative to that of the untreated macrophages (Fig. 1B).

### Effects of CSE on other macrophage characteristics

**Figure 2:**
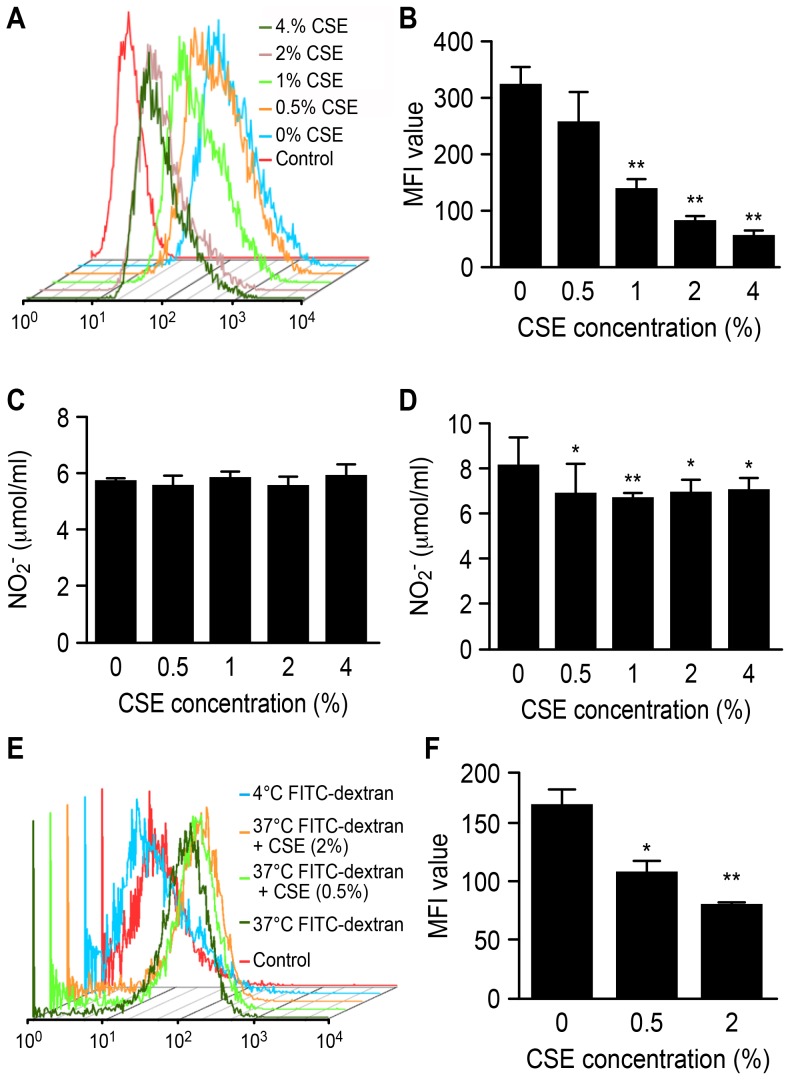
Effects of CSE on Ana-1 macrophage function. **A**, **B** effect of CSE on intracellular ROS level in Ana-1 cells; **C**, **D** NO level of Ana-1 cells treated with CSE for 24 h and 48 h, respectively; **E**, **F** FITC-dextran internalization ability of Ana-1 cells treated with CSE, n = 4, relative to group of cells untreated with CSE; *p<0.05, **p<0.01, ***p<0.001.

M1 macrophages usually produce a certain quantity of ROS to perform their functions, such as destroying invading pathogens, killing tumor cells and removing foreign materials. Conversely, M2 macrophages produce lower ROS levels. The results showed that the intracellular ROS levels in the Ana-1 cells significantly declined following treatment with CSE (p<0.01) (see Figs. 2A and 2B).

High NO production is also a characteristic of M1 macrophages. A significant decrease in the NO level was not observed for the Ana-1 cells that were treated with CSE for 24 h, whereas the NO levels were inhibited by CSE after 48 h (Fig. 2D). These findings also indicate that macrophages could be polarized toward the M2 phenotype by CSE.

FITC-dextran internalization ability is also related to the macrophage phenotype. The analysis results showed that the phagocytic ability of Ana-1 cells declined after CSE treatment (see Figs. 2E and 2F) (0.5% CSE, p<0.05; 2% CSE, p<0.01).

### Effects of CSE on primary mouse peritoneal macrophages, BMDMs and lung alveolar macrophages

**Figure 3:**
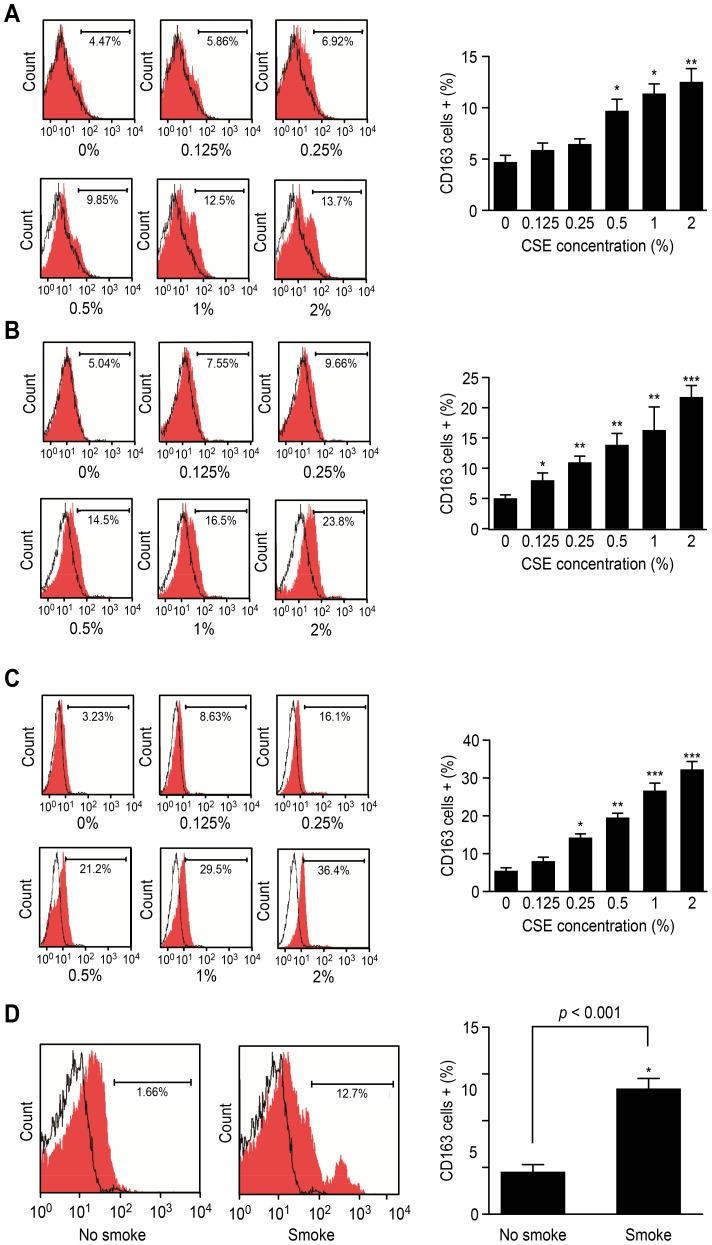
Expression of CD163 marker after mouse macrophages were treated with CSE. **A** CD163 expression in PMs treated with CSE for 3 days, n = 4; **B** CD163 expression in BMDMs treated with CSE for 3 days, n = 4; **C** CD163 expression in lung alveolar macrophages treated with CSE, n = 4; **D** CD163 expression on surface of lung alveolar macrophages from mice completely exposed to CS for 6 days, n = 5, vs. control group; *p<0.05, **p<0.01, ***p<0.001.

Although the M2 polarization of the Ana-1 cell line by CSE was explored, it was necessary to estimate the effects of CSE on primary macrophages. Figures 3A, 3B and 3C show that the number of CD163^+^ cells of all of the 3 types of primary macrophages, PMs, BMDMs and lung alveolar macrophages, significantly increased after CSE treatment for 3 days for CSE levels that were not less than 0.5% for the PMs, 0.125% for the BMDMs and 0.25% for the lung alveolar macrophages. This result further confirmed that CSE could polarize macrophages toward the M2 phenotype.

### *In vivo* effects of cigarette smoke on lung alveolar macrophages of mice

An animal study was conducted on BALB/c mice, and flow cytometry was used to detect their lung alveolar macrophages. After the mice were exposed to CS for 6 days, the number of CD163^+^ lung alveolar macrophages significantly increased over that of the control mice that were not exposed to smoke (p<0.01) (Fig. 3D).

### Effects of CSE on cytokine and enzyme mRNA levels in Ana-1 cells

**Figure 4:**
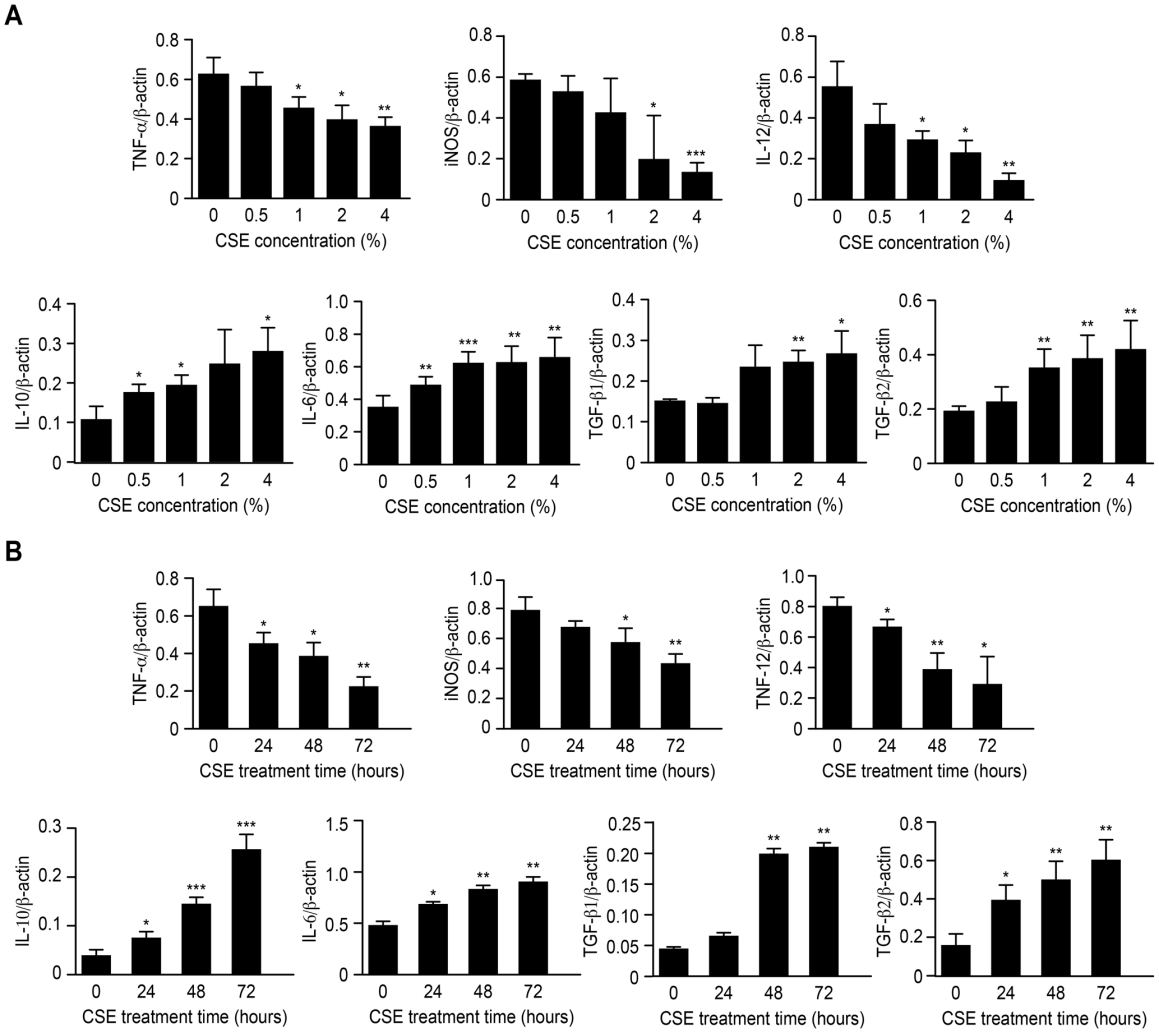
mRNA levels of cytokines expressed by M1/M2 macrophages. **A** mRNA levels of cytokines expressed by Ana-1 cells treated with different CSE concentrations for 48 h, n = 4; **B** mRNA levels of cytokines expressed by Ana-1 cells treated for different times with 2% CSE, n = 4, vs. control group; *p<0.05, **p<0.01, ***p<0.001.

The effects of CSE on macrophage polarization were further evaluated using RT-PCR analysis to measure the expression of M1/M2 cytokines and enzyme. TNF-α, IL-12p40 and iNOS mRNA were measured as inflammatory factors produced by M1 macrophages, whereas IL-10, IL-6, TGF-β1 and TGF-β2 were assessed as M2-related cytokines. The cytokine expression variation was evaluated for different CSE doses, and the results showed that the mRNA levels of the M1 cytokines and enzyme, TNF-α, iNOS and IL-12, gradually declined and those of the M2-related IL-10, IL-6, TGF-β1 and TGF-β2 increased significantly along with the CSE concentration (Fig. 4A).

Likewise, the effects of different treatment times for a CSE dose of 2% on the mRNA expression of M1/M2 cytokines and enzyme were investigated. All of the mRNA levels, except for iNOS, showed significant changes after 24 h of treatment. When the treatment time reached 48 h, the mRNA levels of iNOS, TNF-α and IL-12 decreased significantly, and the IL-10, IL-6, TGF-β1 and TGF-β2 expression gradually increased with the treatment time (Fig. 4B). That is, after treatment with CSE, the mRNA levels of the M1 cytokines in the macrophages significantly declined relative to those of the untreated cells depending on the dose and time, whereas the mRNA level of the M2 cytokines increased significantly (Fig. 4).

### Protein levels of cytokines secreted from macrophages

**Figure 5:**
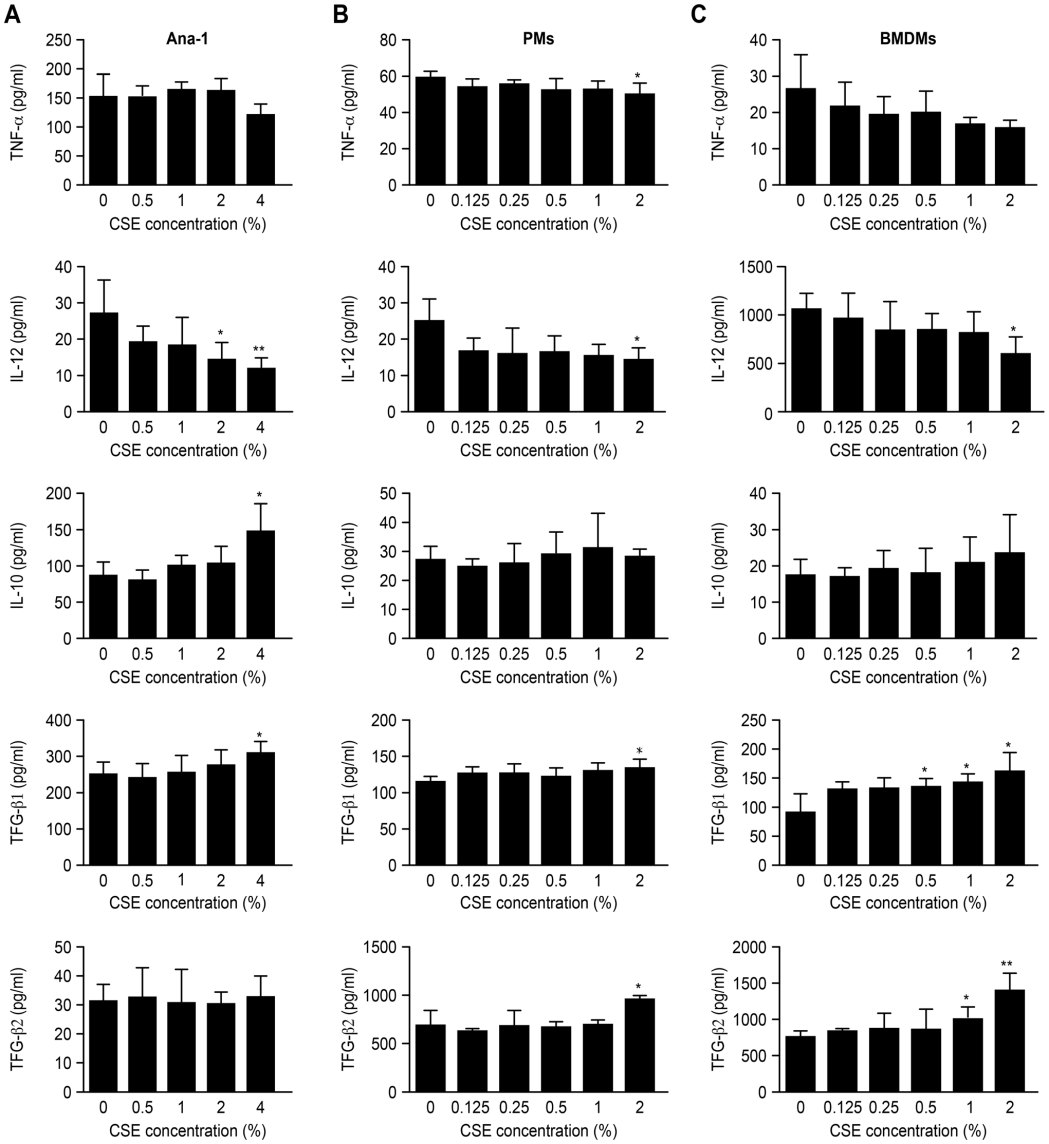
Different cytokines secreted by macrophages in culture supernatant. **A** cytokines released by Ana-1 cells in culture supernatant, n = 4; **B** cytokines released by PMs in culture supernatant, n = 4; **C** cytokines released by BMDMs in culture supernatant, n = 4, vs. control group; *p<0.05, **p<0.01.

The quantity of protein for TNF-α, IL-12, IL-10, TGF-β1 and TGF-β2 that was secreted from different types of macrophages was evaluated. The culture supernatants were collected and detected by ELISA after CSE treatment. The results showed that CSE increased the production of M2 macrophage cytokines above and decreased the levels of M1 related cytokines below those of the untreated controls. In particular, the IL-12 levels in all of the three types of macrophages were significantly lower than those of the controls (2% CSE, p<0.05), whereas the TGF-β1 level was significantly higher (p<0.05) than those of the controls when the CSE concentration reached 4% for the Ana-1 cells, 2% for the PMs and 0.5% for the BMDMs (Fig. 5).

### Effects of CSE on the JAK2-STAT3 pathway

**Figure 6:**
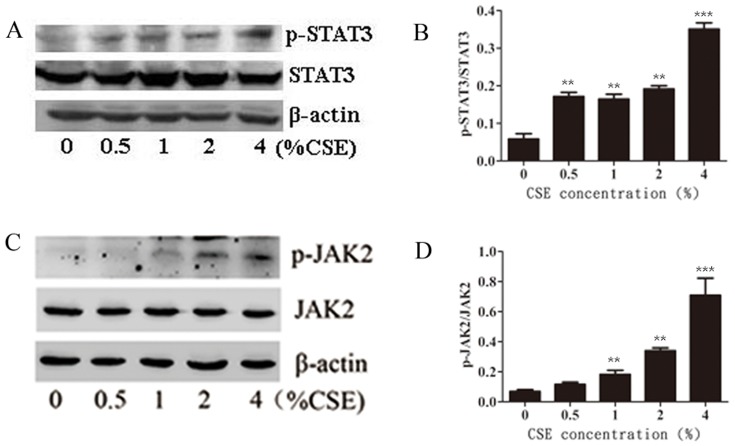
Effects of CSE on activation of JAK2/STAT3 pathway. **A, B** Phosphorylation levels of STAT3 after CSE treatment. **C, D** Phosphorylation levels of JAK2 after CSE treatment. n = 4. *p<0.05, **p<0.01, ***p<0.001, vs control group.

The JAK2-STAT3 signaling pathway is involved in macrophage polarization. Thus, the effects of CSE on the phosphorylation levels of JAK2 and STAT3 in the Ana-1 cells were analyzed by Western blotting. Figure 6 shows that the phosphorylation of both JAK2 and STAT3 in the Ana-1 cells was stimulated by CSE treatment. The statistical analysis showed that the relative levels of p-STAT3/STAT3 and p-JAK2/JAK2 in the CSE-treated cells were significantly higher than those in the untreated cells (p<0.05).

### Blocking of M2 polarization by the JAK2-STAT3 pathway inhibitor WP1066

**Figure 7:**
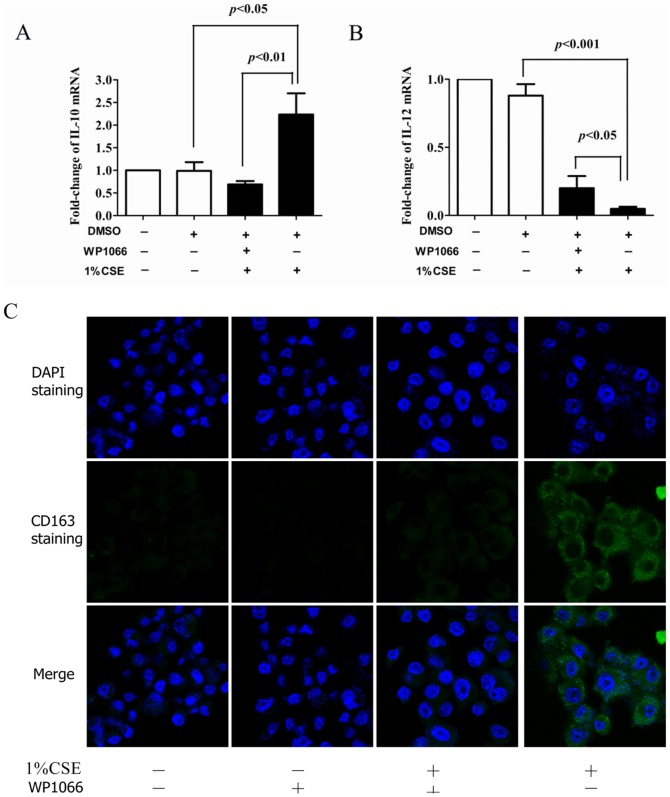
Effects of inhibitor WP1066 on polarization of Ana-1 cells induced by CSE. **A** mRNA level of IL-10 treated with CSE in presence or absence of WP1066; **B** mRNA level of IL-12 treated with CSE in presence or absence of WP1066; **C** confocal laser micrographs of Ana-1 cells cultured with CSE pretreated with or without WP1066; FITC was used to stain cell nuclei for C163 and DAPI.

To confirm the role of the JAK2-STAT3 signaling pathway in macrophage M2 polarization, Ana-1 cells were treated in the presence or absence of the STAT3 phosphorylation inhibitor WP1066 (3 µM) for 1 h prior to treatment with 1% CSE for 48 h. The vehicle DMSO was used as a control. The IL-12 and IL-10 mRNA expression levels were detected by quantitative real-time RT-PCR. The results showed that DMSO did not affect the IL-10 and IL-12 mRNA expressions. When treated with WP1066, the macrophages counteracted the CSE-induced increase in IL-10 (Fig. 7A) and significantly increased the CSE-inhibited expression of IL-12 (Fig. 7B).

The expression of the CD163 marker was inhibited after the Ana-1 cells were treated with WP1066 when observed under a confocal microscope (Fig. 7C).

## Discussion

Macrophages in lungs primarily reside on the respiratory epithelial surface and are exposed directly to the external environment. These macrophages play a central role in defending the lung against pathogens and other environmental hazards and mediate damage and repair in the lung parenchyma [17].

Macrophages are classified along what could be considered a line scale in which M1 macrophages represent one extreme, and M2 macrophages represent the other extreme. The M1 macrophage is primarily responsible for destroying invading pathogens, tumor cells, and foreign materials [18]. These macrophages usually produce high NO levels and reactive oxygen intermediates [19]. Sustained NO production endows macrophages with cytostatic or cytotoxic activity against viruses, bacteria and tumor cells [20]. ROS are also critical components of the antimicrobial repertoire of macrophages [21]. In this study, we found that CSE reduced the macrophage production of NO and ROS, indicating that CSE stimulated the macrophages toward the M2 phenotype. CSE also inhibited the FITC-dextran uptake capacity of macrophages, indicating that the reduction in the phagocytic ability could be related to the M2 macrophages.

M2 macrophages specifically express higher levels of the scavenger receptor CD163, which functions as an anti-inflammatory signal and is a characteristic marker of the M2 macrophage phenotype [22]. Macrophages from patients with respiratory diseases and some healthy smokers have a mixed phenotype and function. These macrophages exhibit significantly impaired efferocytosis, reduced expression of M1 markers involved with antigen presentation (MHC Classes I and II) and increased CD163 levels [23], [24].

Mouse Ana-1 cells, peritoneal macrophages, BMDMs and pulmonary alveolar macrophages expressed higher CD163 levels after CSE treatment than those of untreated controls, verifying that CD163 was markedly expressed on the surfaces of the macrophages from smokers. These results, in combination with findings that macrophages exhibited decreased dextran uptake ability, intracellular ROS levels and nitrate concentrations, indicated that the macrophage injury caused by CSE could weaken the ability of macrophages to kill exotic “invaders” and clear the body's apoptotic cells and residues, which are the most likely initial reasons for M2-polarization.

The M1 phenotype produces IL-12 and TNF-α, whereas M2 macrophages typically produce IL-10, IL-1 receptor antagonist (IL-1ra) and the type II IL-1 decoy receptor [19]. CSE-treated macrophages had low levels of TNF-α, IL-12 p40, and iNOS and high levels of IL-10 and IL-6 that were accompanied by TGF-β secretion depending on the dose and time, which are the characteristics of M2 polarization. All of these observations indicated that the examined macrophages' phenotypes moved in the direction of the M2 activation profile. Cells with this profile demonstrate reduced expression of pro-inflammatory cytokines and increased expression of anti-inflammatory cytokines.

STAT3 activation is related to angiogenesis, cell survival, immunosuppression and tumor invasion in lung cancer [25]–[27]. Animal studies have also demonstrated that STAT3 signaling in macrophages is involved in immune response regulation [28], [29].

The STAT3 pathway is more highly activated in response to several cytokines, including IL-1β, IL-4 and IL-10 in tumor-associated macrophages of the M2 phenotype than in M1 macrophages [30], [31]. Additionally, the major role of the inflammatory cytokine IL-6 is not as an inflammatory mediator but as an activator of the JAK2/STAT3 signaling pathway. Recent studies have also shown that sustained STAT3 activation is mediated by IL-6 in ovarian carcinoma, cholangiocarcinoma, colon cancer and lung adenocarcinoma [32]–[35]. Moreover, STAT3 has a dual role in IL-6 mediated signaling. STAT3 activation may increase IL-6 expression, which in turn may lead to STAT3 phosphorylation, resulting in diverse biological responses [36]. Macrophages from STAT3 knockout mice release higher levels of pro-inflammatory cytokines [37]. Thus, STAT3 is regarded as one of the primary signaling molecules for macrophage polarization toward the M2 phenotype [38]–[41]. In this study, CSE treatment for 1.5 h exerted an apparent activating effect on the phosphorylation of JAK2 and STAT3 in macrophages. Pre-incubation with the inhibitor WP1066 prior to CSE treatment down-regulated a CSE-induced increase of the IL-10 expression, and the expression of IL-12 was stimulated again. This result revealed that the JAK2/STAT3 signaling pathway activation was related to cigarette-smoke-induced macrophage M2 polarization.

In conclusion, our findings clearly show that CSE can markedly induce macrophage M2-polarization to different degrees through the reduced expression of pro-inflammatory cytokines and the increased expression of anti-inflammatory cytokines in macrophages. JAK2/STAT3 activation is a possible mechanism for M2 polarization and may partially account for the initiation, progression and deterioration of various smoking-related inflammatory diseases.
